# New Insights Into Vertigo Attack Frequency as a Predictor of Ischemic Stroke

**DOI:** 10.3389/fneur.2020.593524

**Published:** 2020-12-16

**Authors:** Dongxu Qiu, Lei Zhang, Jun Deng, Zhiwei Xia, Jingfeng Duan, Juan Wang, Rongsen Zhang

**Affiliations:** ^1^Department of Neurology, Xiangya Hospital, Central South University, Changsha, China; ^2^The Third Clinical Medical School of Xiangya, Central South University, Changsha, China; ^3^Department of Geriatrics, The Third Hospital of Changsha, Changsha, China; ^4^Department of Infectious Diseases, Xiangya Hospital, Central South University, Changsha, China; ^5^Department of Ultrasonography, Second Xiangya Hospital, Central South University, Changsha, China

**Keywords:** vertigo attack frequency, ischemic stroke, vascular risk factors, potential stroke, vertigo symptom

## Abstract

**Background:** Recurrent attacks of vertigo account for 2.6 million emergency department visits per year in the USA, of which more than 4% are attributable to ischemic infarction. However, few studies have investigated the frequency of attacks of vertigo before an ischemic stroke.

**Methods:** We conducted this retrospective analysis and manually screened the medical records of 231 patients who experienced recurrent attacks of vertigo prior to an ischemic stroke. Patients were divided into four different groups based on the frequency of vertigo attacks as well as the region of ischemic infarction. Those with ≤2 attacks of vertigo preceding the ischemic stroke were defined as the low-frequency group. Those with ≥3 attacks were defined as the high-frequency group. Clinical parameters, including vascular risk factors, average National Institutes of Health Stroke Scale (NIHSS) score, and infarction volume, were compared between the groups.

**Results:** On analysis, we found that patients with posterior infarction in the high-frequency group exhibited a higher prevalence of vertebral artery stenosis. However, the incidence of diabetes mellitus (DM) was higher in the low-frequency group. In addition, patients with posterior infarction in the low-frequency group were more active in seeking medical intervention after an attack of vertigo. Notably, the brain stem, especially the lateral medullary region, had a higher probability of being involved in posterior infarction in the high-frequency group. However, the cerebellum was more commonly involved in posterior infarction in the low-frequency group.

**Conclusions:** Our findings indicate that the clinical parameters, including arterial stenosis, DM, and magnetic resonance imaging (MRI) findings, differed between the low- and high-frequency groups. We also found that patients in the low-frequency group were more willing to seek medical intervention after the attacks of vertigo. These findings could be valuable for clinicians to focus on specific examination of the patients according to the frequency of vertigo attacks.

## Introduction

Stroke is currently the second leading cause of death worldwide (1, 2). Prompt medical intervention can prevent a transient ischemic attack (TIA) from evolving into acute stroke (3, 4). Recurrent attacks of vertigo have been reported to be important predictors of a future stroke. A recent study reported that vertigo is one of the most common symptoms of vertebrobasilar ischemia, which comprises about 20% of all ischemic strokes. Even more disconcerting are findings from the OxVasc study, which reported that 22% of the patients with posterior circulation stroke reported frequent attacks of vertigo in the 3 months preceding the stroke (5). Importantly, if diagnosed in time, recurrent vertigo symptoms provide a critical opportunity to promptly identify and treat the cause to prevent a stroke. However, vertigo symptoms are common manifestations seen in various conditions, including benign paroxysmal positional vertigo (BPPV) and vestibular migraine. In addition, most patients are asymptomatic with normal findings on neurologic examination after the vertigo attack (6–9), and the symptoms usually dissipate in a short time, even without treatment. Due to these reasons and the absence of sensitive diagnostic tools, it is difficult to distinguish potential ischemic stroke from “peripheral” disorders. However, failure in recognizing the vertigo attack results in worse outcomes as the opportunity to avoid a potential stroke is missed. Therefore, it is important to understand the clinical characteristics including the frequency and duration of the vertigo attacks preceding the ischemic infarction. Nonetheless, no studies investigating these factors have been conducted. We conducted a retrospective analysis and screened the medical records of 231 patients who experienced recurrent vertigo attacks prior to an ischemic stroke. Our findings focused on the differences in the frequency of vertigo prior to an ischemic stroke and the differences in clinical characteristics between the low- and high-frequency groups in both anterior and posterior infarctions. A better understanding of the frequency of vertigo attacks can provide valuable guidance to improve the diagnostic accuracy of a potential ischemic stroke.

## Methods

### Study Design and Population

The clinical data for this retrospective study were collected from the medical records of 1,610 consecutive patients hospitalized with vertigo as the main complaint in Xiangya Hospital and the Second Xiangya Hospital between January 2014 and December 2018. Finally, 231 patients who experienced recurrent vertigo attacks prior to an ischemic stroke were included in the study. Patients were divided into four different groups based on the frequency of vertigo attacks as well as the region of the ischemic infarct. Patients with ≤2 vertigo attacks preceding the ischemic stroke were defined as the low-frequency group. Those with a vertigo attack ≥3 times were defined as the high-frequency group. The patients were divided into four different groups as follows: anterior infarction and low-frequency group, posterior infarction and low-frequency group, anterior infarction and high-frequency group, and posterior infarction and high-frequency group. The study was approved by the ethics committee of Xiangya Hospital. Each patient in the study signed an informed consent document.

### Definition of Acute Ischemic Stroke

All the patients involved in the study were probed by a specially trained neurologist about whether they had a history of recurrent vertigo attack within 2 months prior to the stroke. Vertigo symptoms were defined based on the criteria defined by the Bárány Society (10, 11). If the patients responded affirmatively, additional questions about the exact time of onset of symptoms, detailed description of the vertigo symptoms, frequency and duration of the vertigo attack, and whether they sought medical treatment for the attacks were asked. In addition, subjects were inquired about the presence of other symptoms of focal neurological deficit, such as facial anesthesia or weakness, when the vertigo symptom arose. The duration of vertigo attack, detailed description of the vertigo symptoms, and accompanying symptoms were analyzed to distinguish the peripheral cause from the central vascular cause. Ischemic stroke was defined as an episode of acute focal neurologic deficits with symptoms lasting for more than 24 h. In addition, acute ischemic infarction corresponding to the current neurological deficits was supported by MRI. Acute ischemic stroke was diagnosed by trained neurologists based on the clinical characteristics, MRI findings, or computed tomography evidence. Neurological status was assessed by a neurologist using the 15-item version of the National Institutes of Health Stroke Scale (NIHSS) score (12).

### Definition of Vascular Risk Factors and the Infarction Volume

The following vascular risk factors were evaluated: hyperlipidemia (triglycerides > 1.71 mmol/L and/or cholesterol > 5.17 mmol/L), diabetes mellitus (DM) (previous diagnosis of DM or currently taking hypoglycemic agents), hypertension (diastolic blood pressure > 90 mmHg and/or systolic > 140 mmHg), or currently taking antihypertensive medications. Carotid and vertebral artery ultrasounds were evaluated by specially trained neurologists. The diagnosis of extracranial arterial stenosis was defined as artery stenosis >50% (13). Smoker was defined as continuous or cumulative history of smoking >9 months and ≥1 cigarette per day (14). Alcohol consumption during the past 3 months was also recorded (the standard alcohol consumption criteria is equivalent to 300 ml of beer or 100 ml of wine). MRI was performed on a GE Signa HDX 3.0T MRI (Fairfield, USA). The sequence of T1- and T2-weighted scans, diffusion-weighted imaging, apparent diffusion coefficient, and fluid-attenuated inversion recovery imaging was obtained for every patient. Diffusion-weighted imaging data were acquired using a fast spin-echo planar imaging sequence with echo time (TE) 77.6, repetition time (TR) 50,000, slice thickness of 5.0 mm, and field of view of 220 × 220 mm. Maximum-intensity projection of a three-dimensional volume was applied for data acquisition and imaging reconstruction. All the procedures involved in this study were performed on a post-processing GE machine (Siemens, Inc., Munich, Germany). Detailed information for further analysis was obtained by two experienced observers to reduce the risk of unconscious bias.

### Statistical Analysis

Statistical analyses in this study were performed using SPSS software (version 23.0; IBM, Chicago, IL, USA). The unpaired *t*-test or Mann–Whitney *U*-test was used for analyzing the continuous variables. The χ^2^-test was used to compare categorical variables. The Wilcoxon test was used if the data involved were not normally distributed, and the results are presented as median (range). Continuous variables with normal distribution are expressed as mean ± standard deviation. A *P* < 0.05 was considered statistically significant for all tests.

## Results

**Figure 1:**
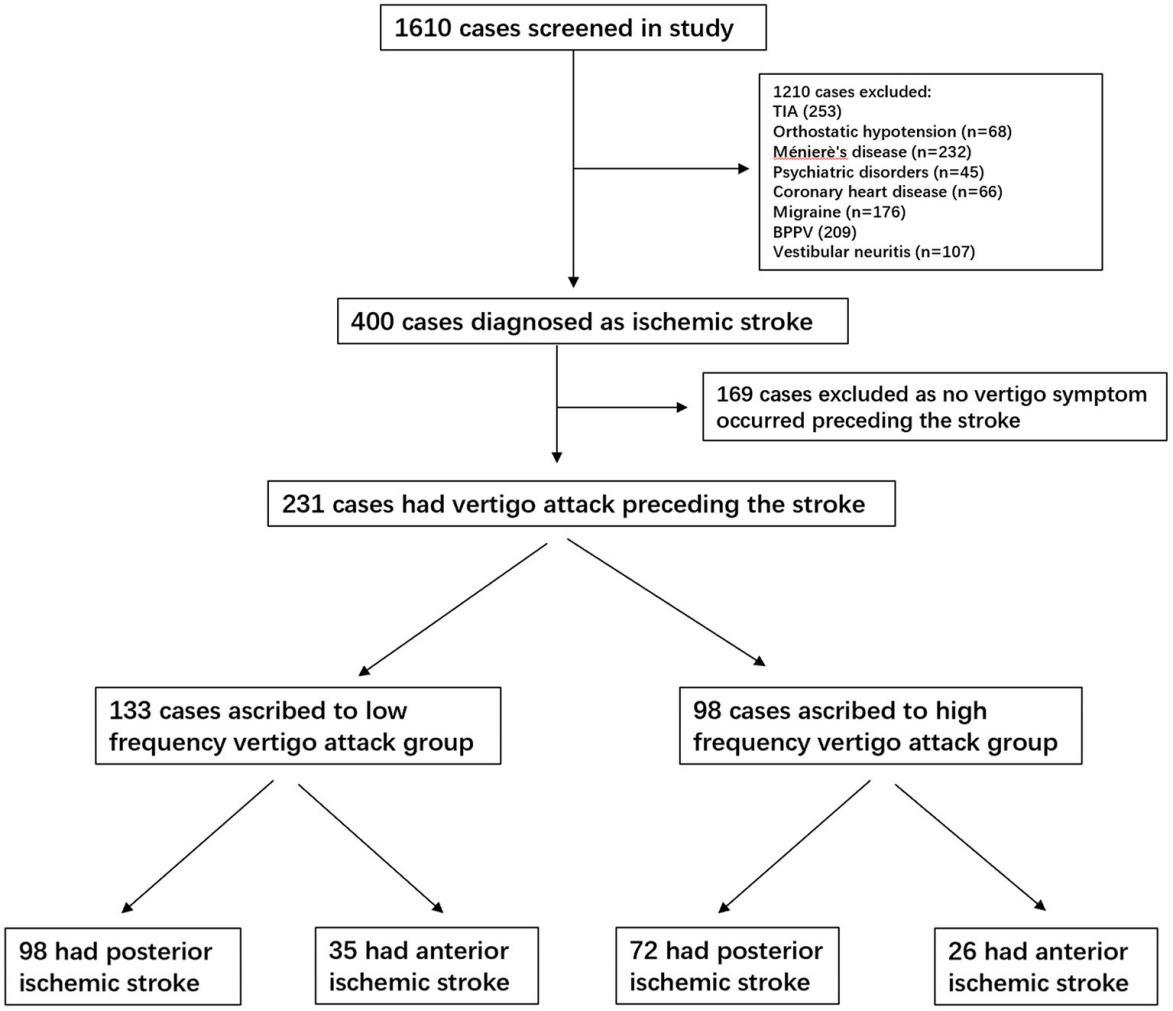
Study flowchart. TIA, transient ischemic attack; BPPV, benign paroxysmal positional vertigo.

We screened all medical records of 1,610 consecutive patients hospitalized with vertigo as the main complaint. Overall, 1,210 patients were diagnosed with Ménierè's disease (*n* = 232), TIA (*n* = 253), BPPV (*n* = 209), vestibular migraine (*n* = 176), vestibular neuritis (*n* = 107), orthostatic hypotension (*n* = 68), coronary heart disease (*n* = 66), labyrinthitis (*n* = 54), and psychiatric disorders (*n* = 45). All these patients were excluded from the analysis. Finally, 231 patients with a definite diagnosis of ischemic infarction and a history of a vertigo attack preceding the stroke were included in the final analysis. Among the cases in the low-frequency group, 98 (42.4%) had involvement of the vertebrobasilar territory (posterior infarction), and 35 (15.1%) had carotid territory involvement (anterior infarction). In the high-frequency group, 72 (31.1%) had involvement of the vertebrobasilar territory and 26 (11.2%) of the carotid territory. Detailed information is presented in the study flowchart (Figure 1).

**Figure 2:**
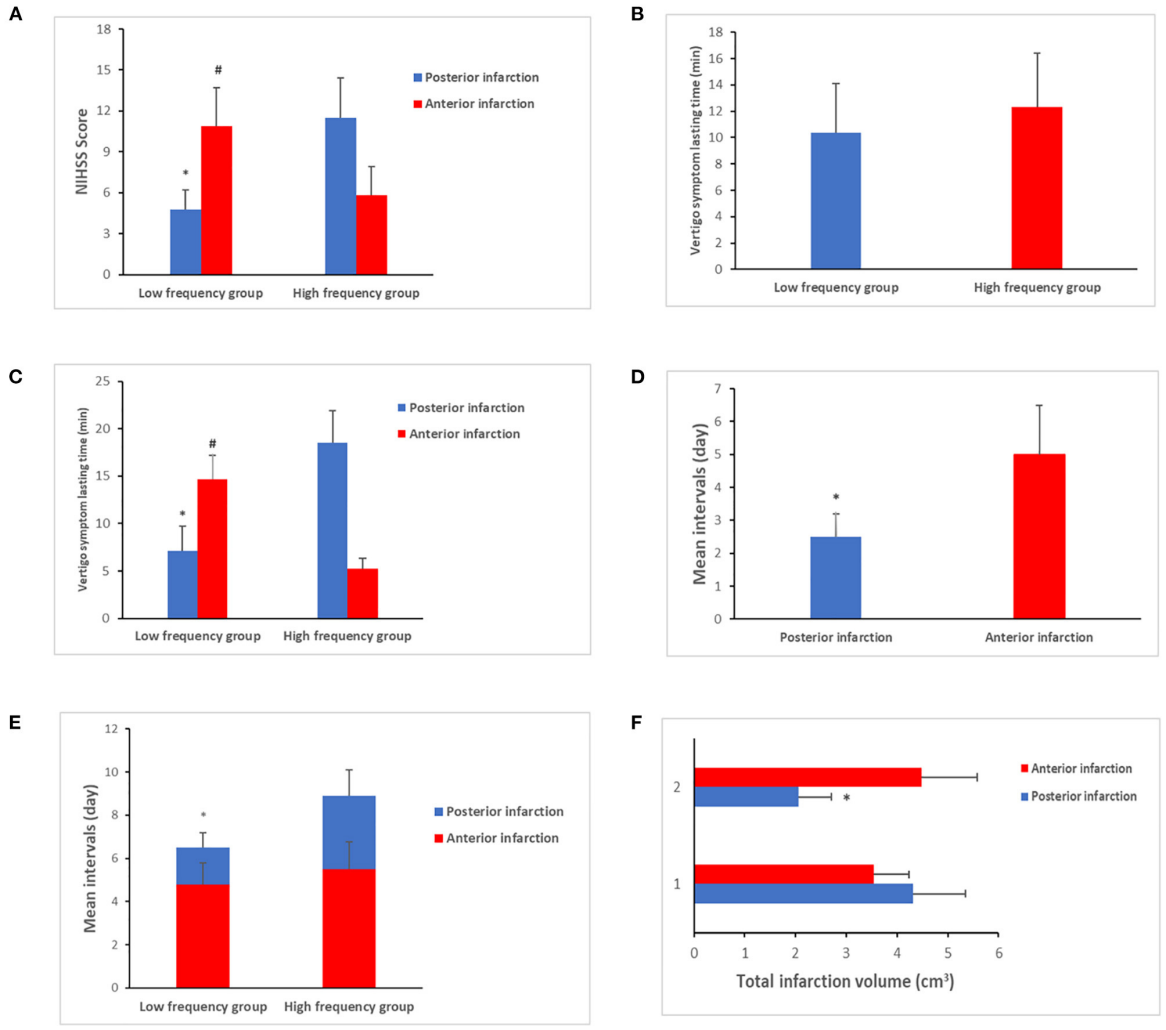
Clinical characteristics comparison between low- and high-frequency vertigo attack groups. **(A)** The average National Institutes of Health Stroke Scale (NIHSS) score was increased in the posterior infarction of the high-frequency group (4.8 ± 1.4 vs. 11.5 ± 2.9, *P* = 0.03), which was decreased in anterior infarction of the high-frequency group (10.9 ± 2.8 vs. 5.8 ± 2.1 *P* = 0.03). **(B)** No differences were exhibited about the vertigo symptom lasting time between the low- and high-frequency groups (10.4 ± 3.7 vs. 12.3 ± 4.1, *P* > 0.05). **(C)** Significance was identified when it comes to the subgroup comparison. As for the posterior infarction cases, the vertigo symptom lasting time tended to be longer in the high-frequency group (7.1 ± 2.6 vs. 18.4 ± 3.4 min, *P* = 0.02). In contrast, anterior infarction cases of the high-frequency group had shorter period of lasting time than the low-frequency group (14.7 ± 2.5 vs. 5.2 ± 1.1 min, *P* < 0.01). **(D)** The mean intervals in patients with posterior infarction were shorter than those of anterior infarction cases (2.5 ± 0.7 vs. 5.0 ± 1.5 days, *P* = 0.03). **(E)** The mean intervals in patients in the high-frequency group were longer than those in patients in the low-frequency group (1.7 ± 0.7 vs. 3.4 ± 1.2 days, *P* < 0.01). In contrast, less patients sought medical care in the high-frequency group (62.9 vs. 30.4%, *P* < 0.01). **(F)** The total infarction volume in the low-frequency group was larger than that in the high-frequency group with a median of 4.32 vs. 2.56 cm^3^ (*P* = 0.02). “*” presents posterior infarction of the high-frequency group compared with posterior infarction of the low-frequency group. “#” presents anterior infarction of the high-frequency group compared with anterior infarction of the low-frequency group.

On comparing the vascular risk factors for posterior infarction between the low- and high-frequency groups, we found that cases in the high-frequency group had a higher prevalence of vertebral artery stenosis (extracranial segment) (9.1 vs. 23.6%, *P* < 0.01). However, the incidence of DM (type II) was higher in the low-frequency group (35.7 vs. 19.4%, *P* = 0.02). In addition, the average NIHSS score increased in the high-frequency group (4.8 ± 1.4 vs. 11.5 ± 2.9, *P* = 0.03) (Figure 2A). Anterior infarction was also compared between the low- and high-frequency groups. Notably, the incidence of internal carotid stenosis (extracranial segment) was also higher in the high-frequency group (11.4 vs. 30.7%, *P* = 0.01) (Table 1). However, the NIHSS score was lower (10.9 ± 2.8 vs. 5.8 ± 2.1, *P* = 0.03) (Figure 2A). Other vascular risk factors, including current smoking, history of hyperlipidemia, hypertension, alcohol, and peripheral vascular disease, did not differ significantly between the groups (Table 1). After age, sex, and body mass index were adjusted, multivariate logistic regression analysis indicated that DM (*P* = 0.03, 95% CI: 2.12–7.96, OR = 4.45) and vertebral artery stenosis (*P* = 0.02, 95% CI: 1.29–5.65, OR = 3.11) were independent risk factors for potential stroke.

**Table 1:** Patient demographics and clinic characteristics.

	**Posterior infarction**			**Anterior infarction**		
	**Low^&^**	**High^#^**	***P***	**Low^&^**	**High^#^**	***P***
Male (*n*, %)	56 (57.1%)	41 (56.9%)	0.28	16 (45.7%)	16 (61.5%)	0.21
Age, Mean ± SD (years)	67.6 ± 7.8	68.2 ± 8.9	0.20	66.9 ± 9.6	70.1 ± 9.2	0.11
Diabetes mellitus (*n*, %)	35 (35.7%)	14 (19.4%)	0.02^*^	9 (25.7%)	8 (30.7%)	0.16
Hypertension (*n*, %)	52 (53.0%)	40 (55.5%)	0.10	18 (51.4%)	11 (42.3%)	0.08
Body mass index, kg/m^2^	25.7 ± 6.3	26.1 ± 5.4	0.24	25.3 ± 5.8	25.8 ± 6.9	0.15
Coronary heart disease (*n*, %)	12 (12.2%)	10 (13.8%)	0.12	7 (18.4%)	5 (19.2 %)	0.13
Internal carotid stenosis (*n*, %)	11 (11.2%)	9 (12.5%)	0.18	4 (11.4%)	8 (30.7%)	0.01^*^
Vertebral artery stenosis (*n*, %)	9 (9.1%)	17 (23.6%)	<0.01^*^	5 (14.2%)	4 (15.3%)	0.08
Peripheral vascular disease (*n*, %)	7 (7.1%)	4 (5.5%)	0.12	3 (8.5%)	3 (11.5%)	0.07
Dyslipidemia (*n*, %)	30 (30.6%)	28 (38.8%)	0.06	11 (31.4%)	9 (34.6%)	0.13
Current smoking (*n*, %)	28 (28.5%)	23 (31.9%)	0.10	9 (25.7%)	7 (26.9%)	0.21
Alcoholism (*n*, %)	18 (18.3%)	15 (20.8%)	0.18	7 (18.4%)	3 (11.5%)	0.06

& *Low frequency of vertigo symptom attack*,

# *High frequency of vertigo symptom attack*.

* *Statistically significant, P < 0.05*.

The duration of vertigo symptoms in the groups was also compared. Despite no differences between the low- and high-frequency groups (10.4 ± 3.7 vs. 12.3 ± 4.1, *P* > 0.05) (Figure 2B), significant differences were observed on subgroup comparison. In cases with posterior infarction, vertigo lasted for a longer duration in the high-frequency group (7.1 ± 2.6. vs. 18.4 ± 3.4 min, *P* = 0.02). In contrast, in cases with anterior infarction, the high-frequency group had a shorter duration of vertigo than the low-frequency group (14.7 ± 2.5 vs. 5.2 ± 1.1 min, *P* < 0.01) (Figure 2C). We also compared the mean intervals (the time from onset of vertigo symptoms to the initiation of therapy). The mean interval in patients with posterior infarction was shorter than that in the patients with anterior infarction (2.5 ± 0.7 vs. 5.0 ± 1.5 days, *P* = 0.03) (Figure 2D). In patients with posterior infarction, the mean interval in the high-frequency group was longer than that in the low-frequency group (1.7 ± 0.7 vs. 3.4 ± 1.2 days, *P* < 0.01). Fewer patients sought consultation for medical care in the high-frequency group after the onset of vertigo (62.9 vs. 30.4%, *P* < 0.01) (Figure 2E).

We also compared the location of infarct between the low- and high-frequency groups. The brain stem, especially the lateral medullary region, was more likely to be affected in posterior infarction in the high-frequency group (20.3 vs. 57.1%, *P* < 0.01). However, in patients with posterior infarction in the low-frequency group, the cerebellum was affected (53.4 vs. 29.8%, *P* = 0.03). In addition, the total area of infarction in the low-frequency group was larger than that in the high-frequency group, with a median of 4.32 vs. 2.56 cm^3^ (*P* = 0.02) (Figure 2F). In cases with anterior infarction, the basal ganglia were more likely to be affected in the low-frequency group (60.4 vs. 31.3%, *P* = 0.02). In contrast, the external cortical zone was more likely to be involved in the high-frequency group (24.3 vs. 42.1%, *P* = 0.04). The above results are presented in Table 2.

**Table 2:** Major findings between low and high frequency vertigo attack group.

	**Posterior infarction**			**Anterior infarction**		
	**Low^&^**	**High^#^**	***P***	**Low^&^**	**High^#^**	***P***
Diabetes mellitus (*n*, %)	35 (35.7%)	14 (19.4%)	0.02	9 (25.7%)	8 (30.7%)	0.16
Internal carotid stenosis (*n*, %)	11 (11.2%)	9 (12.5%)	0.18	4 (11.4%)	8 (30.7%)	0.01
Vertebral artery stenosis (*n*, %)	9 (9.1%)	17 (23.6%)	<0.01	5 (14.2%)	4 (15.3%)	0.08
Vertigo symptom lasting time (min)	7.1 ± 2.6	18.4 ± 3.4	0.02	14.7 ± 2.5	5.2 ± 1.1	<0.01
Mean intervals (day)	1.7 ± 0.7	3.4 ± 1.2	<0.01	4.8 ± 0.5	5.3 ± 0.7	0.34
Patient seek for medical care (*n*, %)	62.9%	30.4%	<0.01	32.6%	38.7%	0.42
Infarction location						
Cerebellum	53.4%	29.8%	0.03	–	–	–
Brain stem	20.3%	57.1%	<0.01	–	–	–
Basal ganglia	–	–	–	60.4%	31.3%	0.02
External cortical zone	–	–	–	24.3%	42.1%	0.04
Total infarction volume(cm^3^)	4.32	2.56	0.02	4.58	5.12	0.08

& *Low frequency of vertigo symptom attack*,

# *High frequency of vertigo symptom attack*.

## Discussion

Ischemic stroke remains the leading cause of disability. Timely medical intervention prevents a TIA from evolving into an acute stroke. Recurrent attacks of vertigo have been reported to be a warning of potential stroke (15–18). However, few studies have investigated the characteristics of the frequency of vertigo attacks prior to the ischemic infarction. Based on the observed results, it was seen that the clinical characteristics between anterior and posterior infarction were different with respect to the frequency of the vertigo attack.

Recurrent vertigo attack remains a clinical challenge, as most patients are asymptomatic with normal findings on neurologic examination after the vertigo attack. In addition, vertigo symptoms are common manifestations seen in other disorders such as BPPV and vestibular migraine. Due to these reasons and the absence of sensitive diagnostic tools, it is difficult to distinguish a potential ischemic stroke from “peripheral” disorders (7–9, 19). We analyzed the clinical characteristics of ischemic stroke based on the differences in the frequency of the vertigo attacks. In a comparison of vascular risk factors, we found that patients with posterior infarction in the high-frequency group had an increased prevalence of vertebral artery stenosis. However, the incidence of DM was higher in the low-frequency group. In addition, the average NIHSS score increased in the high-frequency group. However, in cases of anterior infarction, the incidence of internal carotid artery stenosis was higher in the high-frequency group. Nevertheless, the NIHSS score decreased in that group, which contrasts with the findings in cases with posterior infarction. In addition to the vascular risk factors, the duration of vertigo symptoms was also compared. Although no differences were observed between the low- and high-frequency groups, statistically significant differences were seen in the subgroup comparison. The results showed that the duration of vertigo was longer in the posterior infarction in the high-frequency group. In contrast, vertigo in the anterior infarction in the high-frequency group had a shorter duration. These results indicate that the vascular risk factors, signs of neurological deficits, and duration of vertigo were different between the low- and high-frequency vertigo groups. These clinical characteristics can help in improving the diagnostic accuracy of potential ischemic stroke.

Prompt medical intervention can reduce the morbidity associated with ischemic stroke (20–22). However, not all patients sought medical consultation for vertigo. In our cohort, patients with posterior infarction were more likely to seek medical care than those with anterior infarction in both the low- and high-frequency groups. Patients with posterior infarction in the low-frequency group were more active in seeking medical intervention than those in the high-frequency group, which was contrary to our expectation. Since the incidence of DM was higher in that group, they had more opportunities to seek consultation with the primary physician for the vertigo attack. Moreover, the symptom duration was longer and more intense in that group, thus making them seek an urgent appointment. It was reported that medical treatment prevented as much as 80% of the subsequent stroke (23). However, due to the reluctance in seeking consultation, it remains a challenge to provide patients with timely medical assistance.

Acute ischemic stroke was strongly associated with occlusion of the vascular territory. Of note, inadequate cerebral perfusion can present as recurrent vertigo (22). Therefore, the characteristics of vertigo might be altered due to impaired perfusion. We evaluated the location of infarction between the low- and high-frequency groups. We found that the brain stem, especially the lateral medullary, was more likely to be inflicted in posterior infarction in the high-frequency group. However, posterior infarction in the low-frequency group more commonly involved the cerebellum. The cerebellum is mainly supplied by the posterior–inferior cerebellar artery (PICA) (24). Therefore, recurrent low-frequency vertigo attacks might be a warning sign of stenosis of the PICA. In contrast, posterior infarction in the high-frequency group was more likely to involve the lateral medulla. Infarction in the lateral medulla usually results from occlusion in branches of the basilar artery, which leads to lateral medullary syndrome (LMS) (25, 26). Of note, one of the major focal neurological signs in LMS was recurrent vertigo attacks. Consistent with a previous report, our data confirmed that patients with posterior infarction also presented with recurrent vertigo symptoms. In addition, our results increase the knowledge about vertigo symptoms, indicating that the incidence of vertigo attacks could be more frequent in cases with lateral medullary infarctions than in cases involving the cerebellum.

There are some limitations to this study. First, we excluded patients with hemorrhagic stroke from the analysis because some patients could not remember the details of vertigo events clearly and were unable to provide accurate information on the history of vertigo attacks. Additionally, in cases where a large area was affected by infarction or if a vital area such as the medulla oblongata was involved, the patients might be in a coma and completely unresponsive. In such cases, we were unable to obtain an accurate history of the vertigo attacks, and such cases were excluded from our study. Second, the proportion of patients with ischemic stroke might have been underestimated. As patients with mild stroke were reluctant to seek medical intervention, the focal neurological signs resolved spontaneously. It should also be acknowledged that the relatively small sample size might have resulted in a selection bias. Finally, although multiple criteria were analyzed in the study, the differential diagnosis between peripheral and vascular causes of vertigo is complex, and it is still not clear whether all the patients enrolled in the study had a vascular cause.

## Conclusion

In conclusion, patients with posterior infarction in the high-frequency group had a higher prevalence of vertebral artery stenosis, and the lateral medullary region was more likely to be affected in this group. The incidence of DM was higher in the low-frequency group. Patients with posterior infarction in the low-frequency group were more active in seeking medical intervention after the onset of vertigo, and the cerebellum was more commonly involved in this group. A better understanding of the characteristics of the frequency of vertigo attacks increases the diagnostic accuracy of a potential ischemic stroke.

## Data Availability Statement

The raw data supporting the conclusions of this article will be made available by the authors, without undue reservation.

## Ethics Statement

The studies involving human participants were reviewed and approved by the study was approved by the ethics committee of Xiangya Hospital. The patients/participants provided their written informed consent to participate in this study. Written informed consent was obtained from the individual(s) for the publication of any potentially identifiable images or data included in this article.

## Author Contributions

DQ and JW drafted the manuscript. JW and RZ designed the study, provided statistical analysis, and supervised the study. ZX, JDe, and LZ critically revised the manuscript for intellectual content. JW and RZ made the final revision of the manuscript. All authors contributed to the article and approved the submitted version.

## Conflict of Interest

The authors declare that the research was conducted in the absence of any commercial or financial relationships that could be construed as a potential conflict of interest.
